# An Immune-Related Gene Pairs Signature for Predicting Survival in Glioblastoma

**DOI:** 10.3389/fonc.2021.564960

**Published:** 2021-03-30

**Authors:** Sheng Wang, Xia Xu

**Affiliations:** ^1^Zhejiang Jinhua Guangfu Hospital, Jinhua, China; ^2^Department of General Medicine, Xiangya Hospital, Central South University, Changsha, China; ^3^Department of Pharmacy, Xiangya Hospital, Central South University, Changsha, China

**Keywords:** immune-related gene, glioblastoma, gene pairs, signature, IRGPs

## Abstract

**Background:** Glioblastoma (GBM) is the frequently occurring and most aggressive form of brain tumors. In the study, we constructed an immune-related gene pairs (IRGPs) signature to predict overall survival (OS) in patients with GBM.

**Methods:** We established IRGPs with immune-related gene (IRG) matrix from The Cancer Genome Atlas (TCGA) database (Training cohort). After screened by the univariate regression analysis and least absolute shrinkage and selection operator (LASSO) regression analysis, IRGPs were subjected to the multivariable Cox regression to develop an IRGP signature. Then, the predicting accuracy of the signature was assessed with the area under the receiver operating characteristic curve (AUC) and validated the result using the Chinese Glioma Genome Atlas (CGGA) database (Validation cohorts 1 and 2).

**Results:** A 10-IRGP signature was established for predicting the OS of patients with GBM. The AUC for predicting 1-, 3-, and 5-year OS in Training cohort was 0.801, 0.901, and 0.964, respectively, in line with the AUC of Validation cohorts 1 and 2 [Validation cohort 1 (1 year: 0.763; 3 years: 0.786; and 5 years: 0.884); Validation cohort 2 (1 year: 0.745; 3 years: 0.989; and 5 years: 0.987)]. Moreover, survival analysis in three cohorts suggested that patients with low-risk GBM had better clinical outcomes than patients with high-risk GBM. The univariate and multivariable Cox regression demonstrated that the IRGPs signature was an independent prognostic factor.

**Conclusions:** We developed a novel IRGPs signature for predicting OS in patients with GBM.

## Introduction

Glioma is the most common and the most aggressive cancer of the central nervous system (1). Grade IV glioma: glioblastoma (GBM) is the major subtype of glioma, accounting for more than 48.6% of glioma cases in the USA (2). Although advanced therapies, like surgery, chemotherapy, targeted therapy, and so on, had been applied for GBM, the 5-year relative survival probability of GBM is only limited to 6.8% (3), and the median lifespan is only 19.2 months (4). Due to these discouraging survival data, the development of more accurate tumor-specific biomarkers of GBM for further constructing novel diagnostic signature and guiding clinical treatment is still a priority in GBM management.

There is emerging evidence showing that the immune system plays an important role in the tumorigenesis, development, and metastasis of cancers (5). Immune cells could identify and eradicate tumor cells by recognizing tumor-associated antigen, a key factor for immune surveillance (6). However, tumor cells could act on the immune system to attenuate the effects of immune surveillance and to promote self-development (7). Immunotherapy, a novel treatment method for tumors based on this theory, has been used for the therapy of many cancers (8). In recent years, two-phase III trials of immunotherapy (NCT02017717 and NCT02617589) have been initiated and raised new hopes for the GBM therapy. Nevertheless, owing to the blood–brain barrier, the central nervous system becomes an immunologically privileged situation, and immune escape challenges the effectiveness of immunotherapy in GBM (9, 10). Therefore, the combination management to achieve more efficacious therapeutic benefits is required for complex immune evasion strategies of GBM.

Abnormal gene expression is a common phenomenon in malignant tumors, which may promote the progression of tumors (11). Recently, omics technology and bioinformatics analysis have created an opportunity to identify novel molecular biomarkers and understand potential mechanisms in many tumors. For example, Zhao et al. (12) identified a six-gene risk signature for the outcome prediction of GBM. Several studies have reported immune-related gene pairs (IRGPs) signature in ovarian cancer (13), liver cancer (14), and colorectal cancer (15) and showed well-predictive accuracy. However, up to now, there is no study reporting an IRGP signature in GBM.

In the last two decades, genetic analyses identified point mutation of the gene: isocitrate dehydrogenase (IDH) was an early and common event in gliomagenesis, especially in Grade II and Grade III gliomas (16, 17). In recent years, GBM was defined as a diffuse astrocytic glioma without IDH-mutation (18, 19). Moreover, IDH-mutation in patients with glioma predicts better overall survival (OS) and influences the infiltration of immune cells and immunity (20–22), which may bias the analysis in the study. Hence, in the present study, we did not enroll the patients with GBM with IDH-mutation. Herein, we collected the gene expression matrix from The Cancer Genome Atlas (TCGA) database (https://cancergenome.nih.gov) and immune-related gene (IRG) set from the ImmPort database (https://www.immport.org/home) to establish IRGPs and an IRGP signature in GBM with IDH-wildtype. Meanwhile, the signature was validated with data from the Chinese Glioma Genome Atlas (CGGA) database (http://www.cgga.org.cn). Then, we explored the relationship between the signature and clinical characteristics. Finally, we proved the predictive effectiveness and accuracy of this signature by comparing the area under the curve (AUC) of this signature with that of clinical indexes.

## Materials and Methods

### Data Source and Pre-processing

The gene expression profile and relevant clinical characteristics were downloaded from the databases of TCGA and CGGA. Patients with follow-up time <1 mouth or lacking complete OS would be removed. Three independent cohorts including 514 cases were gathered in the present study [160 cases in Training cohort (TCGA-GBM); 260 cases in Validation cohort 1 (CGGA-693); and 94 cases in Validation cohort 2 (CGGA-301)]. In addition, 472 IRGs were collected from the ImmPort database (https://www.immport.org/home). All raw data from CGGA were transformed with log_2_.

### Establishment of an IRGP Signature

As described in the previous study (23), we performed the pairwise comparison in all samples. Score 1 represented that the expression level of first IRG was higher than that of second IRG in a specific IRGP, and score 0 represented the opposite. Additionally, an IRGP would be deserted if the score of which was 0 or 1 in over 80% of samples. Then, in the Training cohort, the univariate regression analysis with *p*-value <0.001 as the threshold was used to preliminary filtrate IRGPs. Later, the least absolute shrinkage and selection operator (LASSO) regression analysis with iteration = 1,000 and 10-fold cross-validations to prevent overfitting were used to screen up the remaining IRGPs. Ultimately, with *p*-value <0.05 as the cut-off criterion, we applied multivariate Cox regression analysis to determine top OS-related IRGPs, which would be used to develop an IRGP signature and immune risk-score formula for calculating risk score. Based on the risk cut-off score: the median value, patients were classified into two subgroups (high- and low-risk groups).

### Evaluation and Validation of the IRGPs Signature

In the same way, the risk score of each case in Validation cohort 1 and Validation cohort 2 was also calculated. The receiver operating characteristic curve (ROC) was carried out to evaluate the predictive capacity of this IRGPs signature by calculating the AUC.

### Difference of Tumor-Infiltrating Immune Cells Between Two Subgroups

As described previously (24), the CIBERSORT algorithm was utilized to assess the landscape of 22 tumor-infiltrating immune cells (TIICs) in the tumor microenvironment. Cases with *p* < 0.05 were selected for further research. The distribution of 22 TIICs between subgroups was also pictured.

### Gene Set Enrichment Analysis

In the Training cohort, the gene set enrichment analysis (GSEA) was used to assess the potential biological pathways related to this IRGPs signature. False discovery rate (FDR) <0.01 was chosen as the cut-off criterion.

### Statistical Analysis

Table 1 presents all clinical and pathological information of patients with GBM in three cohorts. For categorical data, the differences among different groups were compared with the chi-square test, whereas, for measurement data, the differences were compared with the *t*-test. The univariate and multivariate Cox regression analyses were used to determine independent prognosis-related factors. Survival difference was compared with the logrank test and shown with the Kaplan–Meier method. All statistical analyses were performed using R 3.6.3 (https://www.r-project.org).

**Table 1:** The baseline characteristics of patients with glioblastoma (GBM) in this study.

**Parameter**	**Training cohort**	**Validation cohort 1**	**Validation cohort 2**
Dataset	TCGA	CGGA	CGGA
Platform	Illumina	Illumina	Agilent
**Gender**			
Female	57 (35.62%)	100 (38.45%)	39 (41.49%)
Male	103 (64.38%)	160 (61.54%)	55 (58.51%)
**Age**			
<50	36 (22.50%)	114 (43.85%)	44 (46.81%)
≥50	124 (77.50%)	146 (56.15%)	50 (53.19%)
**KPS**			
≤70	34 (21.25%)	NA	NA
>70	87 (54.38%)	NA	NA
NA	39 (24.37%)	260 (100%)	94 (100%)
**PRS_type**			
Primary	147 (91.88%)	162 (62.31%)	86 (91.49%)
Recurrent	13 (8.12%)	98 (37.69%)	8 (8.51%)
**Subtype**			
Mesenchymal	NA	NA	59 (62.77%)
Non-Mesenchymal	NA	NA	35 (37.23%)
NA	160 (100%)	260 (100%)	NA
**Radiotherapy**			
No	15 (9.38%)	25 (9.62%)	10 (10.64%)
Yes	137 (85.62%)	215 (82.69%)	77 (81.91%)
NA	8 (5.00%)	20 (7.69%)	7 (7.45%)
**Chemotherapy**			
No	NA	40 (15.38%)	34 (36.17%)
Yes	NA	199 (76.54%)	55 (58.51%)
NA	160 (100%)	21 (8.08%)	5 (5.32%)
**Survival status**			
Alive	29 (18.13%)	46 (17.69%)	13 (13.83%)
Dead	131 (81.87%)	214 (82.31%)	81(86.17%)
**Risk score**			
Low	80 (50.00%)	94 (36.15%)	18 (19.15%)
High	80 (50.00%)	166 (63.85%)	76 (80.85%)
Total	160 (100%)	260 (100%)	94 (100%)

*TCGA, The Cancer Genome Atlas; CGGA, Chinese Glioma Genome Atlas; KPS, Karnofsky; NA, information not available*.

## Results

### Establishment of the IRGPs Signature

**Figure 1:**
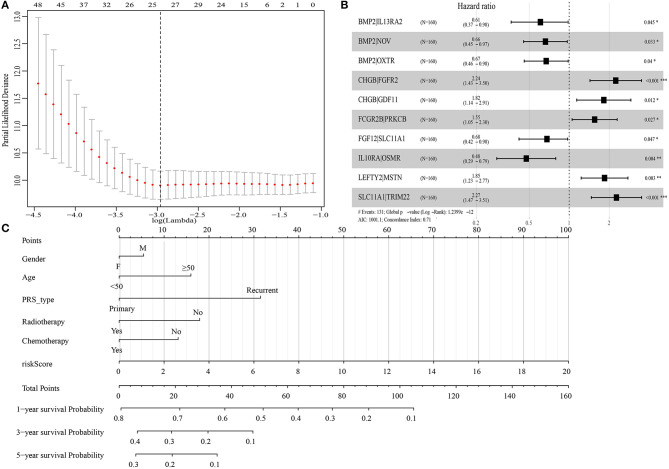
Construction of immune-related gene pairs (IRGPs) signature. **(A)** “Leave-one-out-cross-validation” for the parameter selection in the least absolute shrinkage and selection operator (LASSO) regression. **(B)** The forest map of multivariate Cox regression analysis. **(C)** The prognostic nomogram for the prediction of 1-, 3- and 5-year overall survival (OS) in glioblastoma (GBM). IRGPs, immune-related gene pairs. *p < 0.05, **p < 0.01, ***p < 0.001.

A total of 19,916 IRGPs with score of 0 or 1 in <80% were paired. With the univariate regression analysis, 48 IRGPs were filtered in the Training cohort. Then, the LASSO regression analysis screened out 24 IRGPs for further analysis (Figure 1A). Finally, 10 IRGPs were identified and used to develop an IRGP signature (Table 2 and Figure 1B). Risk score = (−0.4987 × Score_BMP2|IL13RA2_) + (−0.4115 × Score_BMP2|NOV_) + (−0.3985 × Score_BMP2|OXTR_) + (0.8071 × Score_CHGB|FGFR2_) + (0.6000 × Score_CHGB|GDF11_) + (0.4410 × Score_FCGR2B|PRKCB_) + (−0.3854 × Score_FGF12|SLC11A1_) + (−0.7438 × Score_IL10RA|OSMR_) + (0.6128 × Score_LEFTY2|MSTN_) + (0.8191 × Score_SLC11A1|TRIM22_). Based on the cut-off risk score (1.089), patients were divided into two subgroups (high- and low-risk groups). The distribution of risk score and survival status of the IRGPs signature in Training cohort were shown in Supplementary Figure 1A. A nomogram combining risk score and clinical characteristics was also constructed for the prediction of 1-, 3-, and 5-year survival probability (Figure 1C).

**Table 2:** Information on the 10 immune-related gene pairs (IRGPs).

**IRG 1**	**Immune processes**	**IRG 2**	**Immune processes**	**Coefficient**
BMP2	Cytokines	IL13RA2	Cytokine receptors	−0.498
BMP2	Cytokines	NOV	Cytokine receptors	−0.411
BMP2	Cytokines	OXTR	Cytokine receptors	−0.398
CHGB	Cytokines	FGFR2	Cytokine receptors	0.807
CHGB	Cytokines	GDF11	TGFb family member	0.600
FCGR2B	BCR signaling pathway	PRKCB	BCR signaling pathway	0.441
FGF12	Cytokines	SLC11A1	Antimicrobials	−0.385
IL10RA	Cytokine receptors	OSMR	Cytokine receptors	−0.743
LEFTY2	TGFb family member	MSTN	Cytokines	0.612
SLC11A1	Antimicrobials	TRIM22	Antimicrobials	0.819

*IRGPs, immune-related gene pairs; IRG, immune-related gene*.

### Evaluation and Validation of the IRGPs Signature

**Figure 2:**
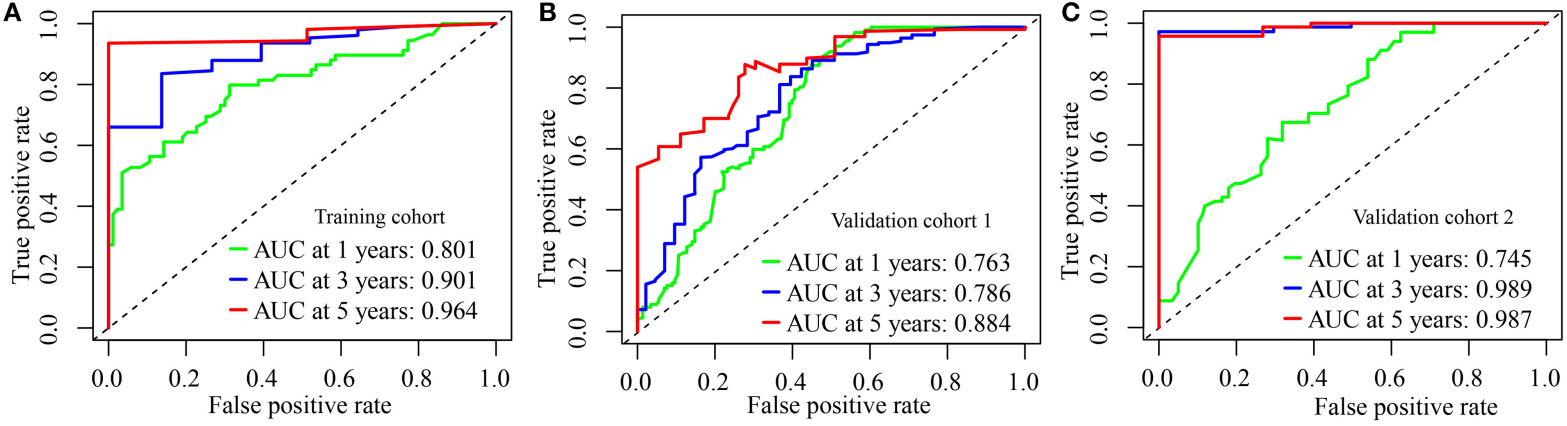
Evaluation of immune-related gene pairs (IRGPs) signature. **(A)** The area under the receiver operating characteristic (ROC) curve (AUC) for 1-, 3-, and 5-year overall survival (OS) of patients with glioblastoma (GBM) in the Training cohort. **(B)** In Validation cohort 1. **(C)** In Validation cohort 2.

We calculated the AUC of this IRGPs signature in the Training cohort to assess the predictive accuracy and effectiveness of the IRGPs signature. The AUC of IRGPs signature in the Training cohort for 1-, 3-, and 5-year OS was 0.801, 0.901, and 0.964, respectively (Figure 2A), which were markedly higher than the AUCs of clinical indexes (Supplementary Figures 2A–C). Also, we calculated the immune risk score of each patient in the Validation cohort 1 and Validation cohort 2 and divided them into high- and low-risk groups according to the cut-off risk score. The distribution of risk score and survival status of the IRGPs signature in the Validation cohort 1 and Validation cohort 2 were shown in Supplementary Figures 1B,C. Then, we validated the predictive accuracy of the IRGPs signature in the Validation cohort 1 and Validation cohort 2. The AUC in Validation cohort 1 reached 0.763 at 1-year, 0.786 at 3-year, and 0.884 at 5-year OS (Figure 2B), and in Validation cohort 2 reached 0.745 at 1-year, 0.989 at 3-year, and 0.987 at 5-year OS (Figure 2C). Similar to the results in the Training cohort, the AUCs of the IRGPs signature at 1-, 3-, and 5-year OS in Validation cohorts 1 and 2 were obviously superior to the AUCs of clinical indexes (Supplementary Figures 2D–I).

Here, we constructed a signature with 10 IRGPs, which included 16 IRGs (BMP2, CHGB, FCGR2B, FGF12, IL10RA, LEFTY2, SLC11A1, IL13RA2, NOV, OXTR, GDF11, PRKCB, FGFR2, OSMR, MSTN, and TRIM22). Among 16 genes, the expressions of 9 genes (CHGB, FCGR2B, IL10RA, SLC11A1, IL13RA2, NOV, OXTR, OSMR, and LEFTY2) in the high-risk group was significantly upregulated, and the expressions of two genes (FGFR2 and MSTN) were opposite (Supplementary Figure 3).

### The IRGPs Signature Is an Important Prognosis-Related Factor

**Figure 3:**
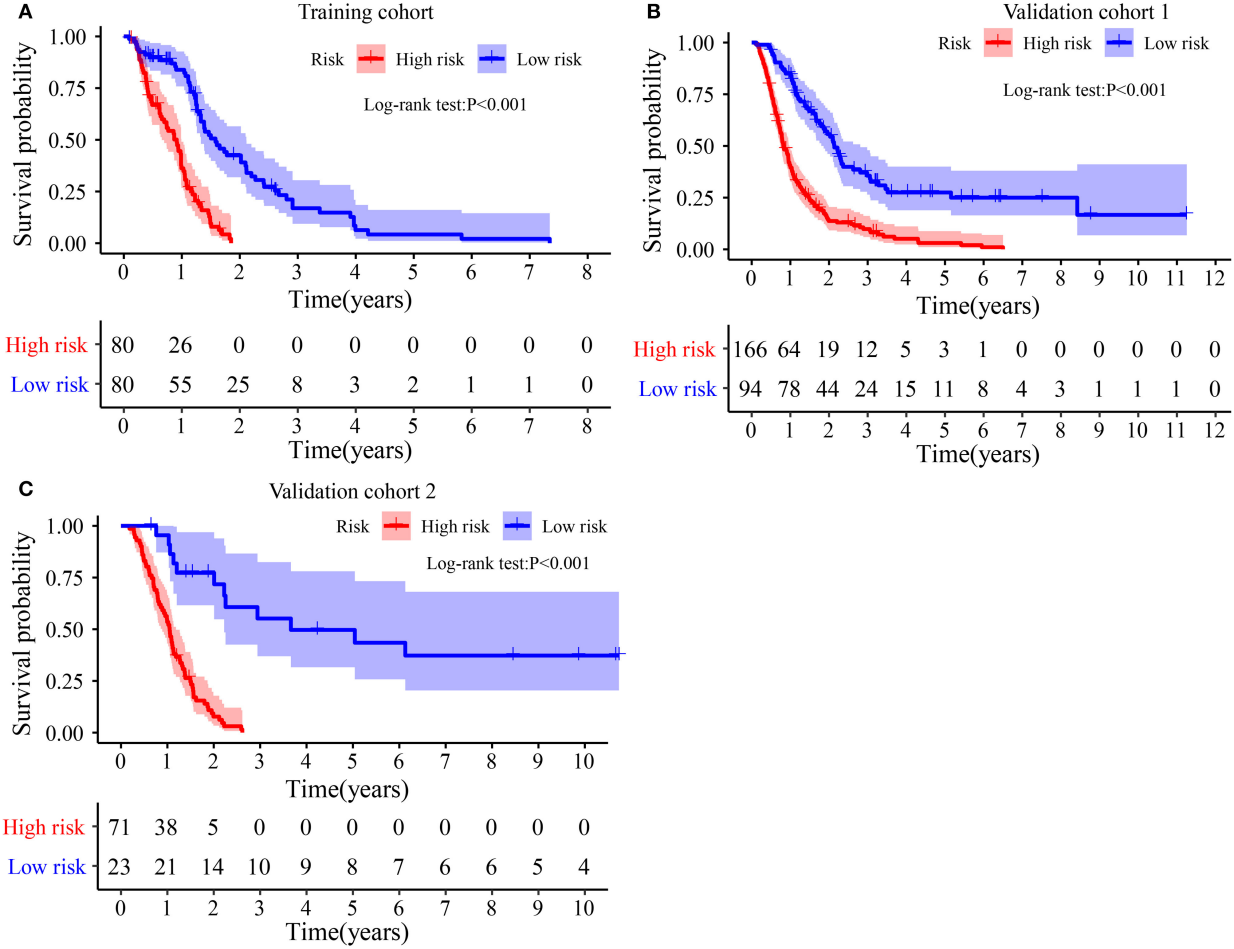
Survival difference between high- and low-risk groups: **(A)** In Training cohort. **(B)** In Validation cohort 1. **(C)** In Validation cohort 2.

**Figure 4:**
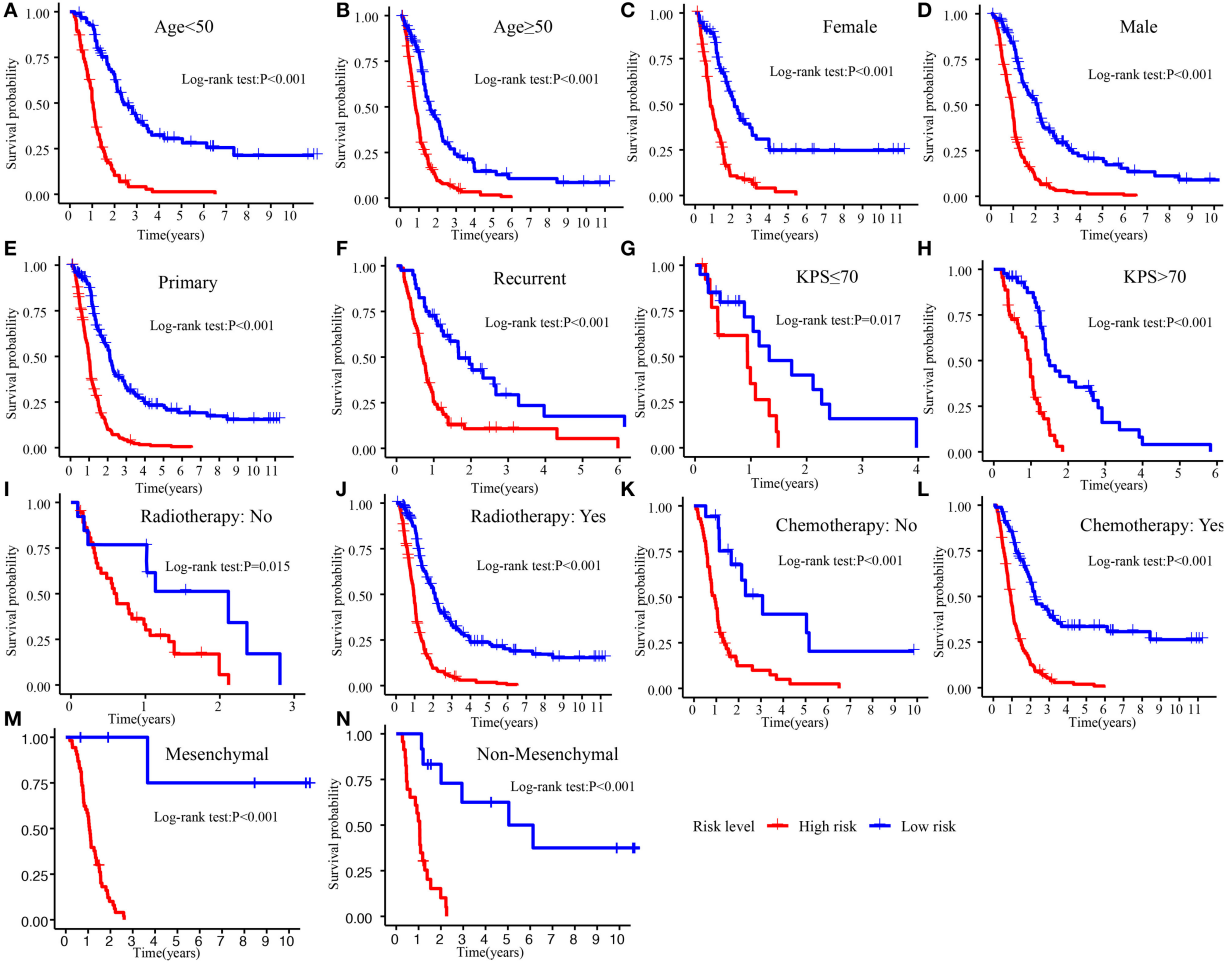
Survival difference between high- and low-risk patients in different subgroup. **(A)** Age <50. **(B)** Age >50. **(C)** Female. **(D)** Male. **(E)** Primary tumor. **(F)** Recurrent tumor. **(G)** KPS ≤70. **(H)** KPS >70. **(I)** Radiotherapy: No. **(J)** Radiotherapy: Yes. **(K)** Chemotherapy: No. **(L)** Chemotherapy: Yes. **(M)** Mesenchymal. **(N)** Non-mesenchymal. KPS, Karnofsky.

The Kaplan–Meier plot in the Training cohort indicated patients with GBM with high-risk scores exhibited worse clinical outcomes than patients with low-risk scores (Figure 3A). Similar results were observed in Validation cohorts 1 and 2 (Figures 3B,C). Moreover, the stratified analysis showed the prognosis of patients with high-risk scores was poorer than that of patients with low-risk scores (Figure 4).

**Figure 5:**
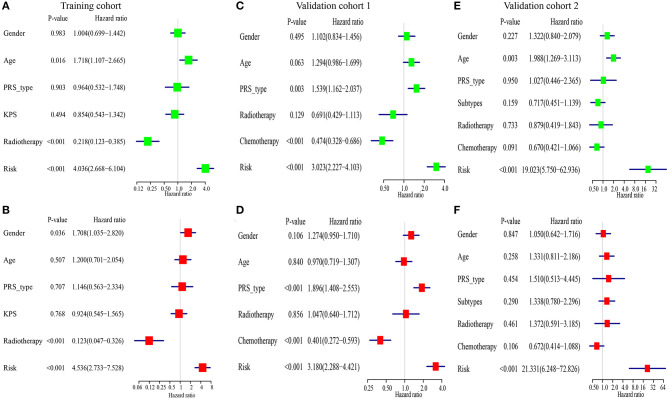
The immune-related gene pairs (IRGPs) signature is an independent prognostic factor of overall survival (OS) of patients with glioblastoma (GBM). **(A,B)** The result of univariable and multivariable Cox regression analysis in the Training cohort. **(C,D)** In Validation cohort 1. **(E,F)** In Validation cohort 2.

For comparing the IRGPs signature with clinical characteristics, we assessed the importance of the IRGPs signature for prognosis of patients with GBM. The univariable Cox regression analysis showed the IRGPs signature was a meaningful factor influencing the prognosis of patients with GBM [Training cohort: HR = 4.036, 95% CI (2.668, 6.104), *p* < 0.001, Figure 5A; Validation cohort 1: HR = 3.023, 95% CI (2.227, 4.103), *p* < 0.001, Figure 5C; Validation cohort 2: HR = 19.023, 95% CI (5.750, 62.936), *p* < 0.001, Figure 5E]. In addition, the multivariable Cox regression analysis indicated that the IRGPs signature was an independent prognosis-related factor [Training cohort: HR = 4.536, 95% CI (2.733, 7.528), *p* < 0.001, Figure 5B; Validation cohort 1: HR = 3.180, 95% CI (2.288, 4.421), *p* < 0.001, Figure 5D; Validation cohort 2: HR = 21.331, 95% CI (6.248, 72.826), *p* < 0.001, Figure 5F].

### Association Between the IRGPs Signature and Clinical Indexes

**Figure 6:**
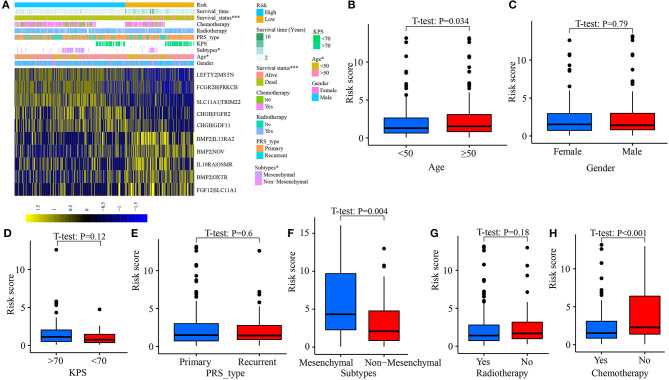
Correlation between risk score and clinical characteristics. **(A)** Heat map for the distribution of clinicopathological features between high- and low-risk groups. **(B)** The difference of risk scores among patients aged <50 and aged ≥50. **(C)** Between female and male. **(D)** Between patients with KPS ≤70 and >70. **(E)** Between primary tumor and recurrent tumor. **(F)** Between different molecular subtypes. **(G)** Between patients with radiotherapy and without radiotherapy. **(H)** Between patients with chemotherapy and without chemotherapy. *p < 0.05, ***p < 0.001.

Clinicopathological data, including age, gender, tumor molecular subtype, KPS (Karnofsky), recurrent, radiotherapy, and chemotherapy, were collected. As shown in Figure 6A, the distribution of age (*p* = 0.047) and tumor subtype (*p* = 0.036) between high- and low-risk groups was significantly different. In addition, the risk scores of patients aged ≥50 were significantly increased (*p* = 0.034, Figure 6B). An analogous phenomenon was observed in patients with mesenchymal GBM (*p* = 0.004, Figure 6F). Compared with patients without chemotherapy, patients treated with chemotherapy had lower risk scores (Figure 6H). However, there was no difference in the risk scores between female patients and male patients (*p* = 0.790, Figure 6C), patients with KPS ≤70 and KPS >70 (*p* = 0.120, Figure 6D), primary tumor and recurrent tumor (*p* = 0.600, Figure 6E), and, patients treated with radiotherapy and without radiotherapy (*p* = 0.180, Figure 6G).

### Relationship Between the IRGPs Signature and TIICs

**Figure 7:**
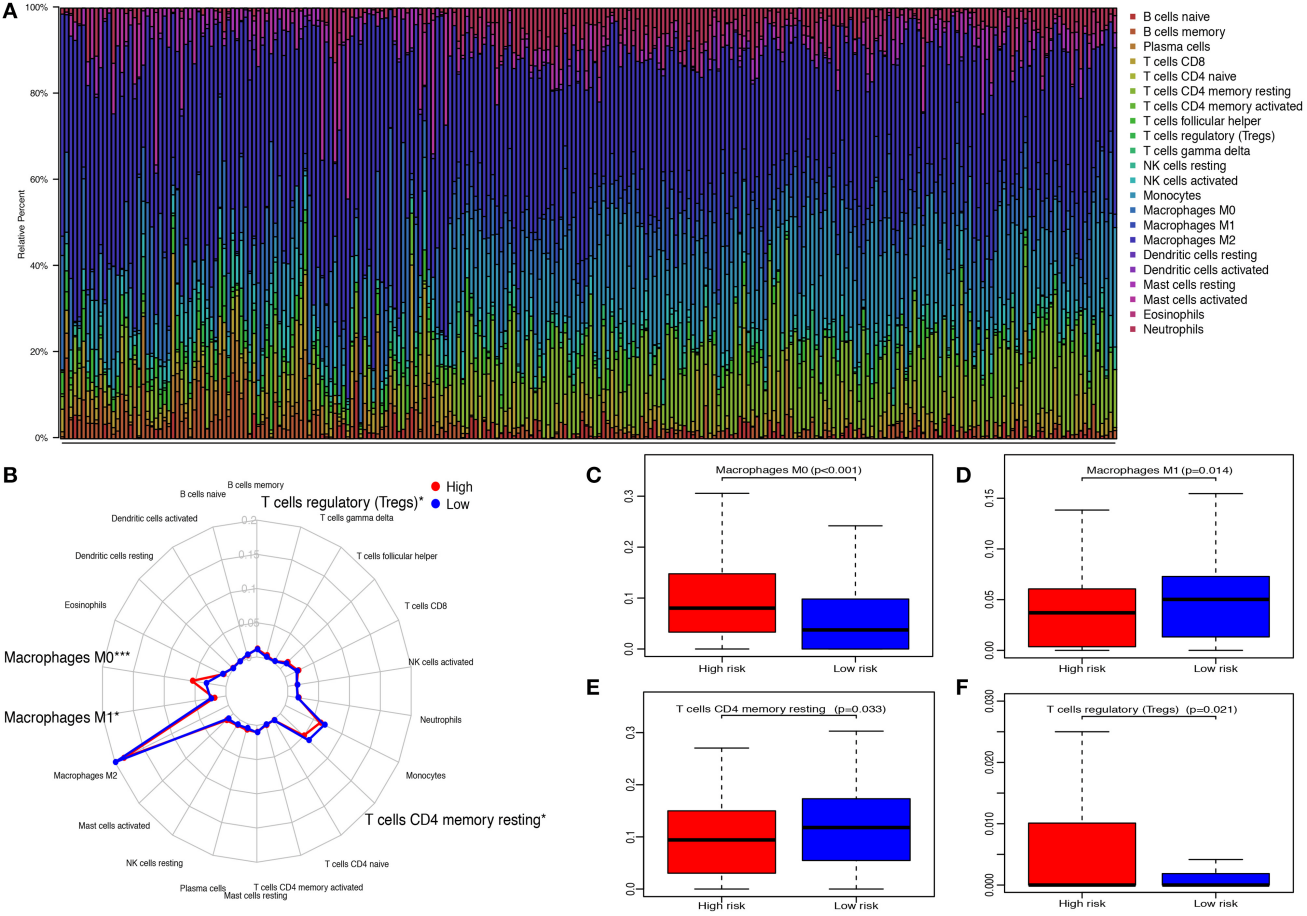
Relationship between immune-related gene pairs (IRGPs) and tumor-infiltrating immune cells (TIICs). **(A)** The proportions of 22 TIICs in 248 samples were quantified by CIBERSORT. **(B)** The difference of 22 TIICs between high- and low-risk groups. **(C)** The difference of macrophages M0. **(D)** The difference of macrophages M1. **(E)** The difference of resting CD4 memory T cells. **(F)** The difference of regulatory T cells (Tregs) between high- and low-risk groups. *p < 0.05, ***p < 0.001.

The CIBERSORT method was applied to quantify the proportions of 22 TIICs. A total of 248 samples (116 low-risk patients and 132 high-risk patients) met the cut-off value: *p* < 0.05. The highest quantity of immune cell among 22 TIICs was macrophages (M0, M1, and M2) (49.73%), followed by monocytes (12.53%) and resting memory CD4 T cells (10.45%) (Figures 7A,B). Compared with high-risk patients, the fractions of macrophages M1 (*p* = 0.014, Figure 7D) and resting memory CD4 T cells (*p* = 0.033, Figure 7E) in low-risk patients were significantly elevated, whereas, the fractions of macrophages M0 (*p* < 0.001, Figure 7C) and regulatory T cells (Tregs) (*p* = 0.021, Figure 7F) were decreased.

Then, we investigated the distributions of TIICs between score:1 and score:0 of 10 IRGPs. As shown in Supplementary Figure 4, we found that the proportion of macrophages M0 in IL10RA|OSMR:0, LEFTY2|MSTN:1, and SLC11A1|TRIM22:1 was increased. The fractions of macrophages M1 and resting memory CD4 T cells in FCGR2B|PRKCB:0, LEFTY2|MSTN:0, and SLC11A1|TRIM22:0 were also increased. Moreover, the content of Tregs in FCGR2B|PRKCB:1, LEFTY2|MSTN:1, SLC11A1|TRIM22:1, BMP2|NOV:0, and FCGR2B|PRKCB:0 was upregulated. In addition, the difference of the distributions of several TIICs between score:1 and score:0 of 10 IRGPs was observed (Supplementary Table 1).

### Gene Set Enrichment Analysis

**Figure 8:**
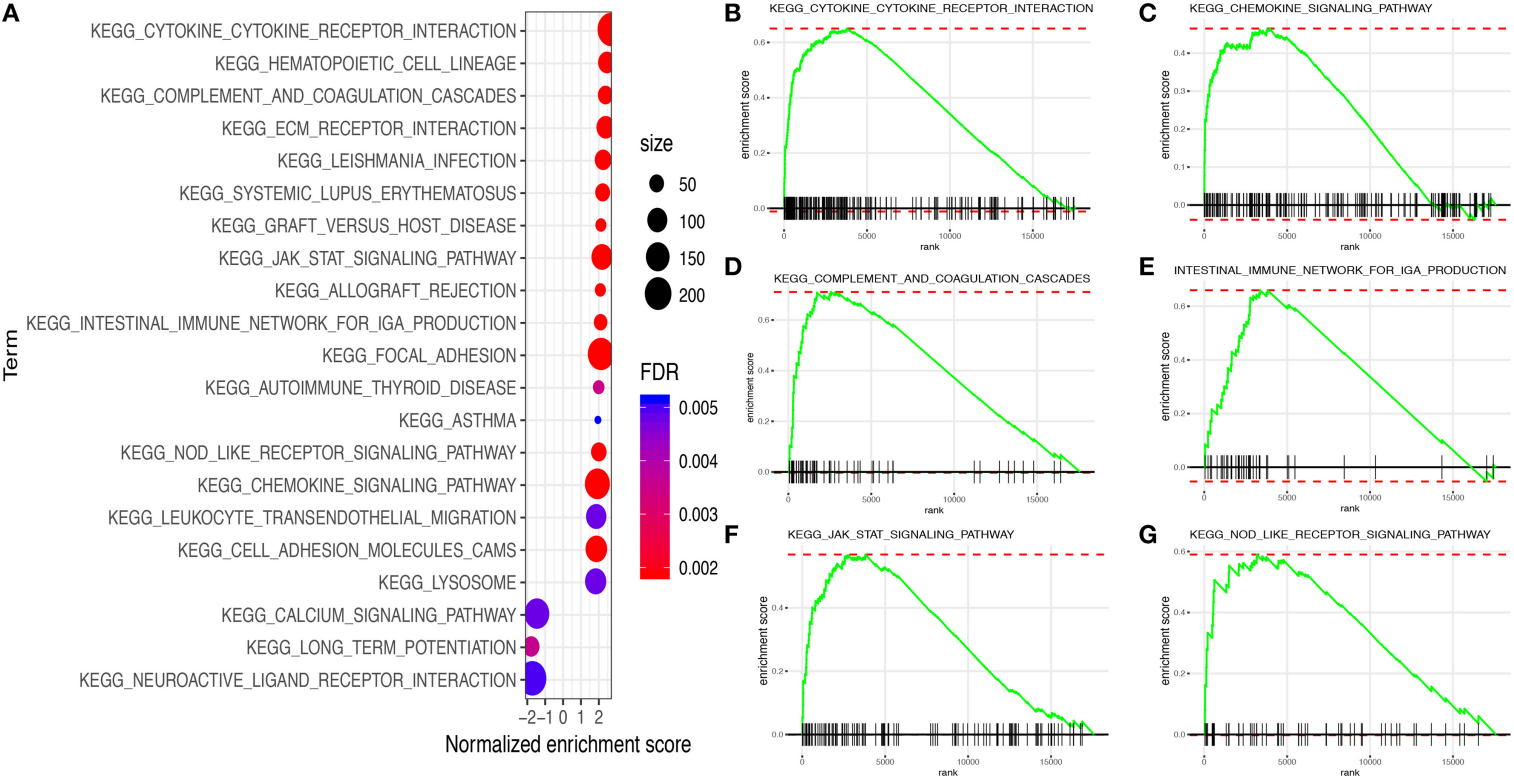
Gene set enrichment analysis (GSEA) between high and low immune risk groups. **(A)** 22 Kyoto Encyclopedia of Genes and Genomes (KEGG) pathway-related gene sets. **(B)** Cytokine–cytokine receptor interaction. **(C)** Chemokine signaling pathway. **(D)** Complement and coagulation cascades. **(E)** Intestinal immune network for IgA production. **(F)** JAK–STAT signaling pathway. **(G)** NOD-like receptor signaling pathway.

The results of GSEA illustrated that a total of 22 Kyoto Encyclopedia of Genes and Genomes (KEGG) pathway-related gene sets were enriched. Among them, a few pathways were closely correlated with the immune system, such as “cytokine–cytokine receptor interaction,” “chemokine signaling pathway,” “Janus Kinase (JAK)-signal transducer and activator of transcription (STAT) signaling pathway,” “complement and coagulation cascades,” “Nucleotide-binding oligomerization domain (NOD)-like receptor signaling pathway,” and “intestinal immune network for IgA production” (Figure 8).

## Discussion

Glioblastoma is the frequently occurring and most aggressive malignancy in the central nervous system (25). The 5-year survival rate of patients with GBM after diagnosis is still limited to 6.8% (3). It is important to seek the tumor-specific markers and build risk stratification for the assessment of prognosis in GBM, which may facilitate the development of novel strategies for the diagnosis and therapy of GBM.

The immune system is a considerable influencing factor for the progression of cancers (5). Immunotherapy as a novel treatment method has revolutionized cancer therapy (8), which also provides new hope for the treatment of GBM. In this study, we downloaded the IRG expression matrix from the TCGA database to establish IRGPs and develop an IRGP signature for the prediction of clinical outcomes of patients with GBM. Based on the cut-off value, patients were segmented into two subgroups. The survival analysis showed that patients with high-risk scores had a more unfavorable prognosis. In addition, the univariable and multivariate Cox regression analysis in three cohorts demonstrated the IRGPs signature was an independent prognostic factor in patients with GBM. However, the HR of univariable and multivariate Cox regression analysis in the Validation cohort 2 was 19.023 and 21.331, respectively, substantially higher than that in the Training cohort and Validation cohort 1. It may be caused by the patients with low-proportion low-risk scores in the Validation cohort 2 (high-risk score:low-risk score = 71: 23).

Later, we estimated the predicted capacity of this IRGPs signature. The AUCs of the IRGPs signature for predicting OS probability in the Training cohort reached 0.800 at 1 year, 0.901 at 3 years, and 0.948 at 5 years, which were significantly superior to the AUCs based on the clinical characteristics, such as age, gender, chemotherapy, recurrent, and so on (Supplementary Figures 2A–C). The AUCs for 1-, 3-, and 5-year OS in the Validation cohorts 1 and 2 were similar to that in the Training cohort and were also obviously higher than that of clinical indexes (Supplementary Figures 2D–I). All data demonstrated that the IRGPs signature was suitable to predict 1-, 3- and 5-year OS in GBM.

Recently, several studies explored prognostic biomarkers and evaluated the prognosis of GBM with immune-related features. For example, Zhang et al. (26) used a random forest algorithm to screen the tumor microenvironment (TME)-related genes and developed a TME score with five genes using the multivariate Cox regression analysis. The Kaplan–Meier plot showed a significant survival difference between patients with high- and low-TME scores. A study utilized the univariable Cox regression analysis and LASSO regression analysis to establish an immune-based prognostic scoring model for predicting survival probability in GBM with the AUC = 0.657 at 1 year, 0.667 at 3 years, and 0.667 at 5 years (27). Another study constructed a Foxp3-related immune prognostic signature in patients with GBM using the LASSO regression analysis (28). The AUC for predicting 1- and 3-year OS was 0.633 and 0.695, respectively. In 2018, Zhou et al. (29) performed the survival analysis to select prognostic immune-related-lncRNA and then carried out the multivariate Cox regression analysis to develop a six immune-related lncRNA prognostic risk model. The AUCs of the model for predicting 5-year OS was 0.842. All of those studies illustrated the well-predictive ability of immune-related features. Different from previous studies, herein, we constructed IRGPs in GBM and applied the univariable Cox regression analysis and LASSO regression analysis to preliminary filtrate IRGPs. Finally, we executed the multivariate Cox regression analysis to further screen and develop IRGPs signature. Up to now, this is the first time using IRGPs, a novel immune-related feature, to build a signature for predicting OS probability in GBM. The AUCs of the signature were obviously superior to that in the study of Qin et al. (27) and Guo et al. (28) and slightly preferable to the AUC in the study of Zhou et al. (29), showing the IRGPs signature is robust and reliable.

The IRGPs signature consisted of 10 gene pairs, including 16 IRGs (BMP2, CHGB, FCGR2B, FGF12, IL10RA, LEFTY2, SLC11A1, IL13RA2, NOV, OXTR, GDF11, PRKCB, FGFR2, OSMR, MSTN, and TRIM22). Between low- and high-risk groups, the expression of 11 genes (CHGB, FCGR2B, IL10RA, SLC11A1, IL13RA2, NOV, OXTR, OSMR, LEFTY2, FGFR2, and MSTN) was with statistical difference (Supplementary Figure 3). Fibroblast growth factor receptor 2 (FGFR2) and fibroblast growth factor 12 (FGF12) are the members of the fibroblast growth factor family, which is involved in the activation of the Kirsten Rat Sarcoma (RAS)-Mitogen-Activated Protein Kinase (MAPK) and the Threonine Kinase (AKT) and the PI3K–AKT pathway, and the cell proliferation, differentiation, and apoptosis of cancers (30). The studies demonstrated that they could influence tumor development *via* regulating macrophages, enhance PD-1 and PD-L1 expression, and promote inflammation (31–34). Fc fragment of IgG receptor IIb (FCGR2B) encoded a receptor for the Fc region of immunoglobulin gamma complexes. It is involved in several regulatory functions, such as modulating B cells producing antibodies and the phagocytosis of immune complexes (35). Moreover, it could bring about the downmodulation of cell activation triggered through regulating antigen receptors on B cells and T cells and upregulation of IgG responses, which contribute to humoral immune responses (35, 36). Protein kinase C beta (PRKCB) is a kind of serine-specific protein kinases, and it is involved in various cellular signaling processes, such as the activation of B cells and cell proliferation (37). Moreover, it also could mediate the differentiation of dendritic cells (37). Tripartite motif-containing 22 (TRIM22) and solute carrier family 11 member 1 (SLC11A1) participate in host resistance to the pathogen and interferon-mediated antiviral effects (38–40). SLC11A1 also participates in the activation of macrophages to promote macrophage immune effector functions, such as inhibiting immune inflammation and altering the processing and presentation of antigens by enhancing the autoimmune T cell (39, 40). TRIM22 could regulate macrophage autophagy (38). Interleukin 10 receptor subunit alpha (IL10RA), interleukin 13 receptor subunit alpha 2 (IL13RA2), and oncostatin M receptor (OSMR) are cytokine receptors and could mediate the immunosuppressive signal and the synthesis of proinflammatory cytokines. Previous studies showed IL10RA was highly related to the clinical outcomes of colorectal cancer and melanoma (41, 42). IL13RA2 is overexpressed in over 75% of patients with GBM and related to prognosis and participates in the downregulation of immune response mediated by helper T cells (43, 44). Immunization with IL13RA2 DNA vaccine could inhibit tumor growth and induces tumor immunity (45). OSMR could transduce signaling induced by oncostatin M, IL6, and IL31 and regulated immune cell-mediated metabolism (46). Basic research showed that OSMR contributed to the adjusting of extracellular matrix process and local immune response in GBM, which was highly associated with the development of GBM (47). Chromogranin B (CHGB), oxytocin receptor (OXTR), and cellular communication network factor 3 (CCN3) also belong to cytokine and cytokine receptor, which play a vital role in the homeostasis of innate and adaptive immunity and remarkably affect anti-tumor immunity through activating cytotoxic T cells and cancer immunotherapy (48, 49). Bone morphogenetic protein 2 (BMP2), myostatin (MSTN), growth differentiation factor 11 (GDF11), and left–right determination factor 2 (LEFTY2) belong to the transforming growth factor-beta superfamily, which has pleiotropic effects on cell proliferation, differentiation, invasion, metastasis, and apoptosis of cancer cells. It also influences inflammation, neurogenesis, and immune response *via* regulating immune cell differentiation and the activity of immune components (50–52).

Nowadays, emerging studies show treatment targeting the tumor microenvironment is an inspiring way to overcome therapeutic escape issues (53). Herein, we depicted the landscape of immune cells in the tumor microenvironment of GBM and found macrophages was the highest proportion immune cell, which was in line with previous findings (53, 54). Furthermore, we identified that the proportion of macrophages M1 in the low-risk group was significantly increased, whereas, macrophages M0 was opposite. After induced by lipopolysaccharide and interferon-γ stimulation, macrophages M0 may polarize into M1 that could promote GBM cell proliferation, invasion, and metastasis (55–57). Additionally, in the high-risk group, the fractions of Tregs were also significantly increased. The accumulation of Tregs in the tumor microenvironment functions as immunosuppression *via* inhibiting proliferation and degranulation of other immune cells (58). Increased proportion of Tregs was highly associated with tumor recurrence and decrease of survival and efficacy of immunotherapeutic strategies in GBM (59).

In this study, the IRGPs signature demonstrated an excellent predictive capability for patients with GBM. However, there are still several limitations that need to be improved. (1) As all cases were obtained from open databases, the possibility of selection bias cannot be excluded. (2) The IRGPs signature was developed based on RNA-seq data and microarray expression. It is costly and time-consuming. (3) Due to all samples were collected from the public database, the potential selection bias could not be eliminated, and some clinical information, such as treatments, molecular subtype, and so on, were missing in some samples, which may result in information bias. Meanwhile, the study was a retrospective study. Hence, validation of prospective samples, especially samples with complete clinical information, was still needed. (4) In the Training cohort, we did not obtain the clinical information: the status of IDH-mutation, which may present in enrolled patients with GBM, especially the patients with recurrent tumor. (5) No experimental studies were performed to testify the finding in this study. Thus, further basic experiments are warranted to validate the finding of this research.

## Conclusions

In conclusion, in this study, we constructed a 10-IRGPs signature for predicting 1-, 3-, and 5-year OS in GBM. The IRGPs signature would be an effective and convenient means to predict the clinical outcome of GBM and provide a novel way for risk assessment.

## Data Availability Statement

Publicly available datasets were analyzed in this study, this data can be found at: The Cancer Genome Atlas (https://portal.gdc.cancer.gov/).

## Author Contributions

SW: data collection, data analysis, and writing. XX: study design, data analysis, and revised the manuscript. All authors reviewed the manuscript.

## Conflict of Interest

The authors declare that the research was conducted in the absence of any commercial or financial relationships that could be construed as a potential conflict of interest.

## Supplementary Material

Supplementary Figure 1 Distribution of risk score and survival status. **(A)** Training cohort, **(B)** Validation cohort 1, **(C)** Validation cohort 2.

Supplementary Figure 2 The area under the curves (AUCs) of clinical indexes for predicting 1-, 3-, and 5-year OS. **(A–C)** in the Training cohort. **(D–F)** in Validation cohort 1. **(G–I)** in Validation cohort 2. AUC, the area under the receiver operating characteristic curve; OS, overall survival.

Supplementary Figure 3 The different expression of 16 immune-related genes (IRGs) between high- and low-risk groups. ^*^*p* < 0.05, ^**^*p* < 0.01, ^***^*p* < 0.001.

Supplementary Figure 4 Comparison of 22 immune cells between 10 IRGPs scores:1 and 0. **(A)** The distributions of Macrophages M0 between score:1 and score:0 of IL10RA/OSMR, **(B)** LEFTY2/MSTN, **(C)** SLC11A1|TRIM22. **(D)** The distributions of Macrophages M1 between score:1 and score:0 of FCGR2B|PRKCB, **(E)** LEFTY2|MSTN, **(F)** SLC11A1|TRIM22. **(G)** The distributions of resting memory CD4 T cells between score:1 and score:0 of BMP2|NOV, **(H)** FCGR2B|PRKCB, **(I)** LEFTY2|MSTN, **(J)** SLC11A1|TRIM22. **(K)** The distributions of Tregs cells between score:1 and score:0 of BMP2|NOV **(L)** FCGR2B|PRKCB, **(M)** LEFTY2|MSTN, **(N)** SLC11A1|TRIM22, **(O)** FCGR2B|PRKCB.

Supplementary Table 1 P-values of Wilcoxon test for the distribution of 22 immune cells between 10 IRGPs score:1 and 0.
