# Supplementary figures and images for: An Immune-Related Gene Pairs Signature for Predicting Survival in Glioblastoma
Source: Front Oncol. 2021 Mar 30;11:564960. doi: 10.3389/fonc.2021.564960 (PMC8042321; doi:10.3389/fonc.2021.564960)

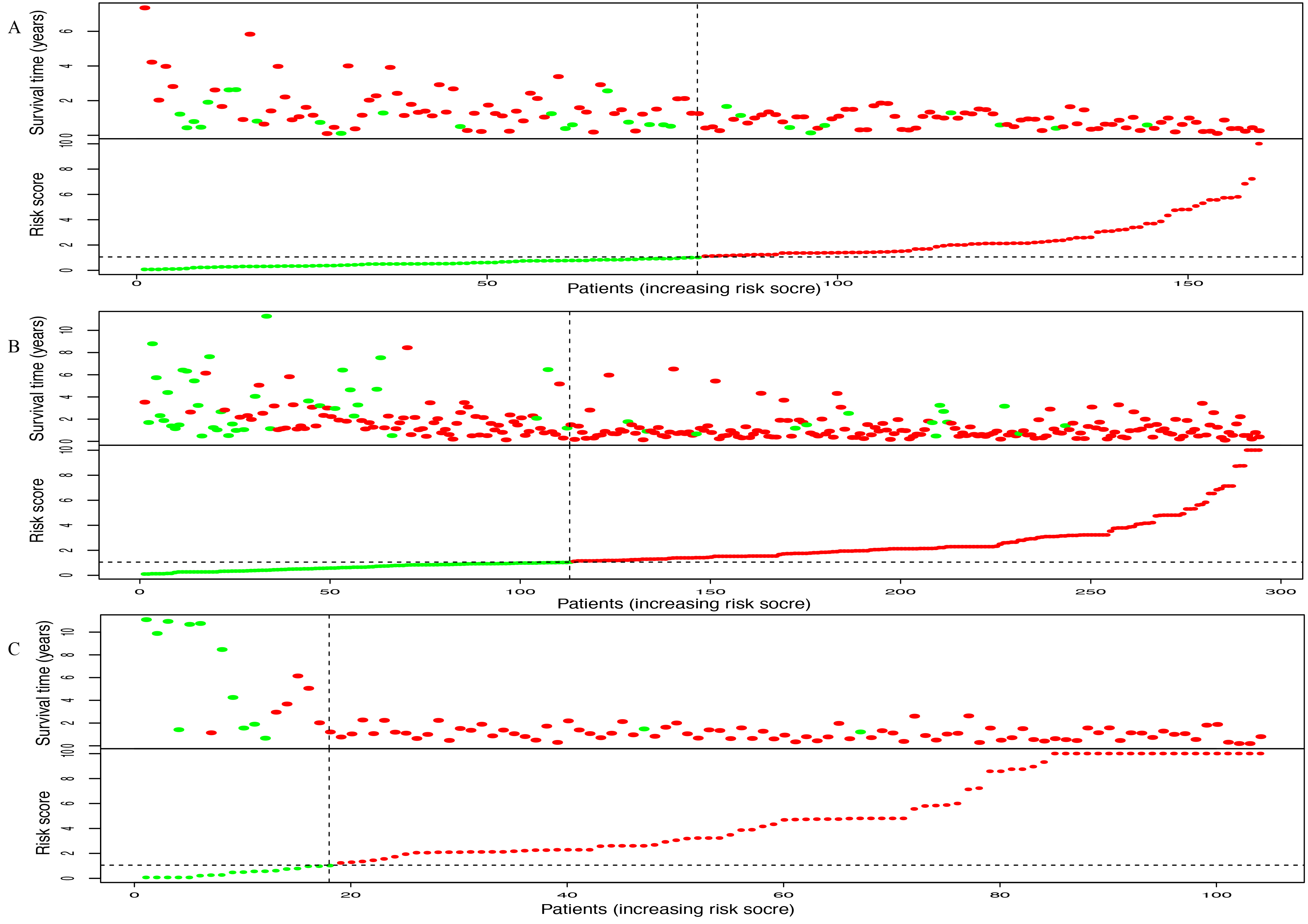

Supplement: Supplementary Figure 1 — Distribution of risk score and survival status. (A) Training cohort, (B) Validation cohort 1, (C) Validation cohort 2. [file Image_1.JPEG]

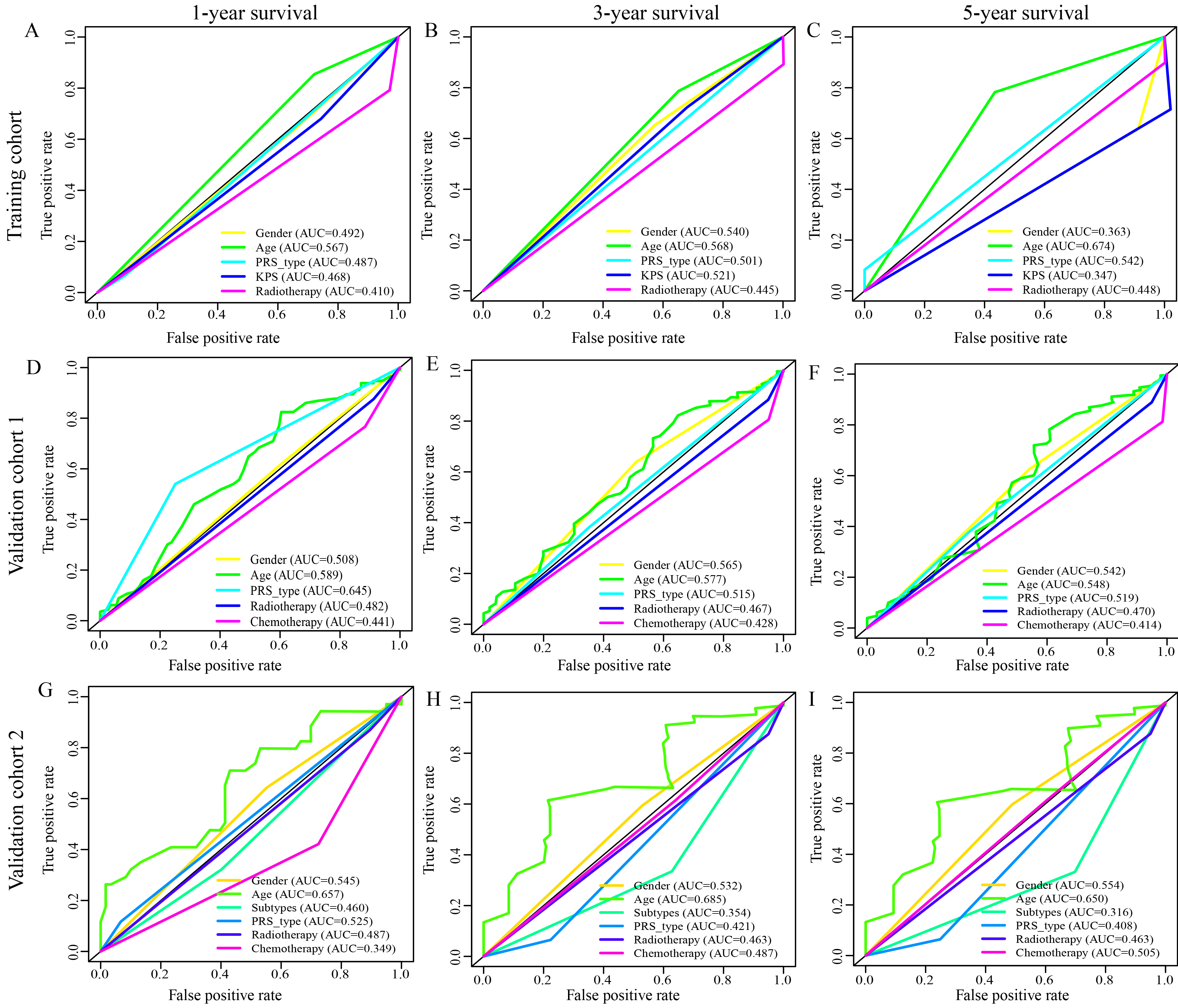

Supplement: Supplementary Figure 2 — The area under the curves (AUCs) of clinical indexes for predicting 1-, 3-, and 5-year OS. (A–C) in the Training cohort. (D–F) in Validation cohort 1. (G–I) in Validation cohort 2. AUC, the area under the receiver operating characteristic curve; OS, overall survival. [file Image_2.JPEG]

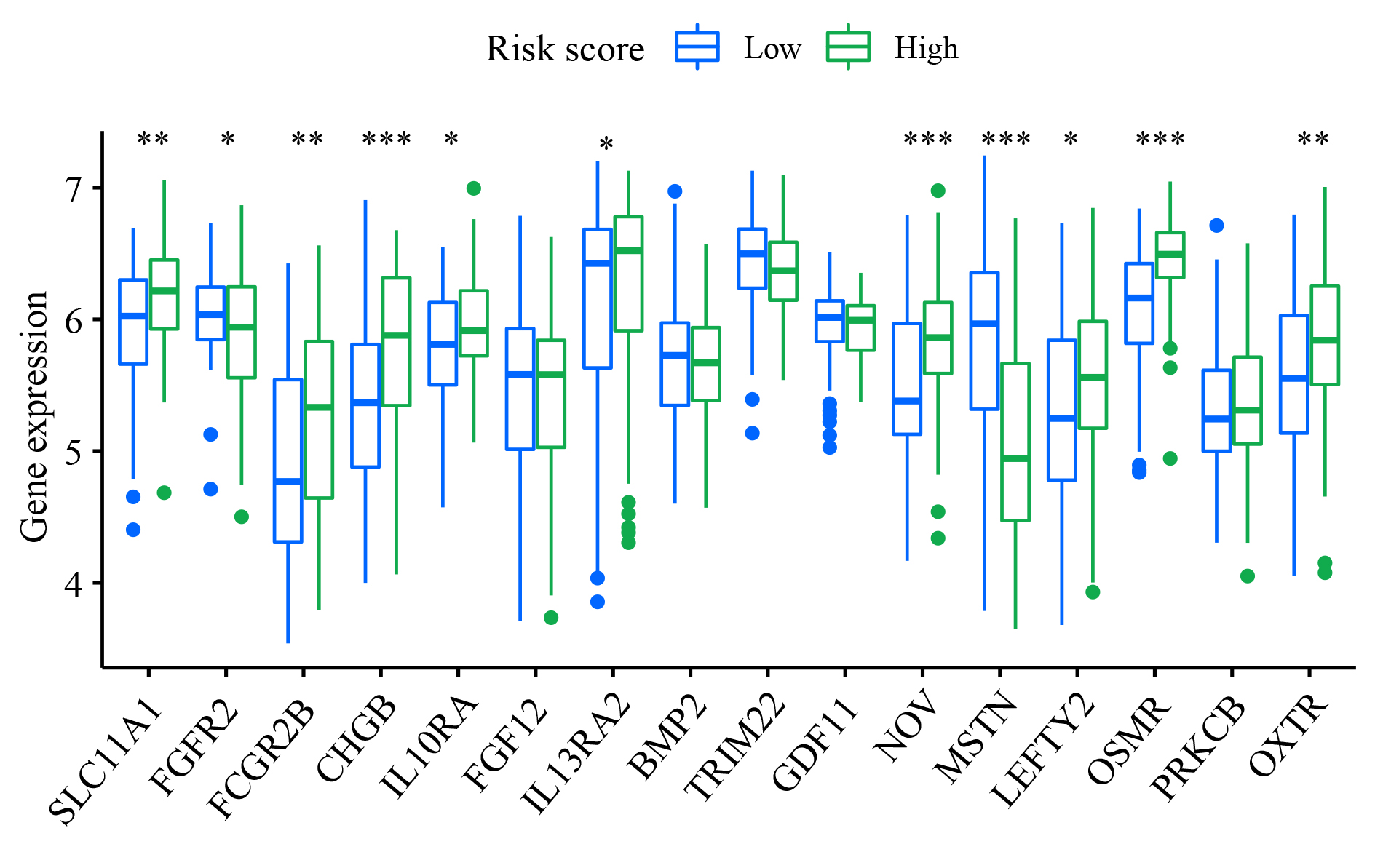

Supplement: Supplementary Figure 3 — The different expression of 16 immune-related genes (IRGs) between high- and low-risk groups. *p < 0.05, **p < 0.01, ***p < 0.001. [file Image_3.JPEG]

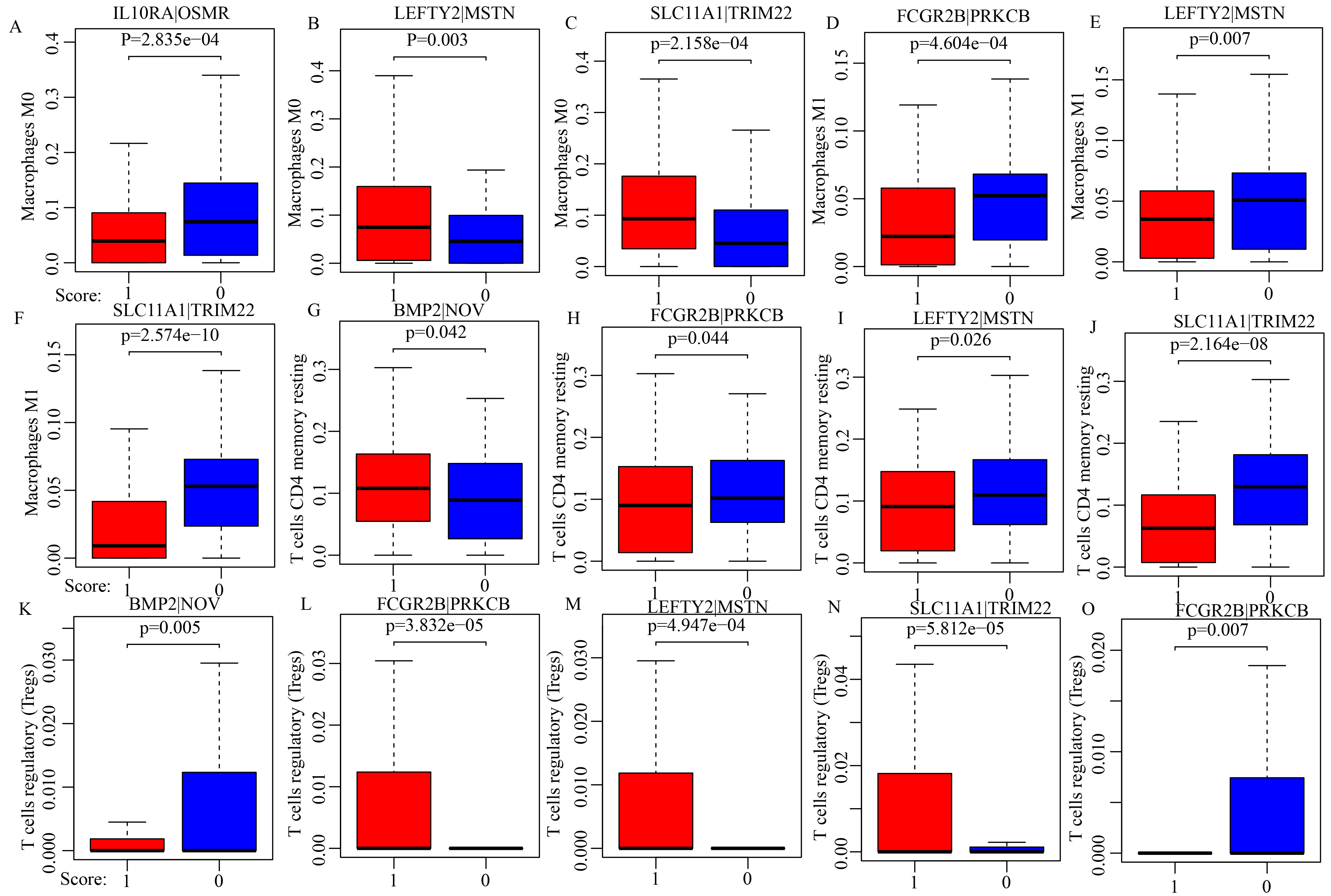

Supplement: Supplementary Figure 4 — Comparison of 22 immune cells between 10 IRGPs scores:1 and 0. (A) The distributions of Macrophages M0 between score:1 and score:0 of IL10RA/OSMR, (B) LEFTY2/MSTN, (C) SLC11A1|TRIM22. (D) The distributions of Macrophages M1 between score:1 and score:0 of FCGR2B|PRKCB, (E) LEFTY2|MSTN, (F) SLC11A1|TRIM22. (G) The distributions of resting memory CD4 T cells between score:1 and score:0 of BMP2|NOV, (H) FCGR2B|PRKCB, (I) LEFTY2|MSTN, (J) SLC11A1|TRIM22. (K) The distributions of Tregs cells between score:1 and score:0 of BMP2|NOV (L) FCGR2B|PRKCB, (M) LEFTY2|MSTN, (N) SLC11A1|TRIM22, (O) FCGR2B|PRKCB. [file Image_4.JPEG]
