# Supplementary material for: An Immune-Related Gene Pairs Signature for Predicting Survival in Glioblastoma
Source: Front Oncol. 2021 Mar 30;11:564960. doi: 10.3389/fonc.2021.564960 (PMC8042321; doi:10.3389/fonc.2021.564960)
Supplement: Supplementary Table 1 — P-values of Wilcoxon test for the distribution of 22 immune cells between 10 IRGPs score:1 and 0. [file Table_1.docx]

Table S1. P-values of Wilcoxon test for the distribution of 22 immune cells between 10 IRGPs score:1 and 0.

|  | BMP2\|  IL13RA2 | BMP2\|  NOV | BMP2\|OXTR | CHGB\|FGFR2 | CHGB\|GDF11 | FCGR2B\|PRKCB | FGF12\| SLC11A1 | IL10RA\|OSMR | LEFTY2\|MSTN | SLC11A1\|TRIM22 |
| --- | --- | --- | --- | --- | --- | --- | --- | --- | --- | --- |
| B cells naive | 0.917 | 0.967 | 0.881 | 0.127 | 0.602 | 0.025 | 0.672 | 0.690 | 0.791 | 0.277 |
| B cells memory | 0.450 | 0.030 | 0.851 | 0.438 | 0.887 | 0.047 | 0.981 | 0.001 | 0.871 | 0.121 |
| Plasma cells | 0.511 | 0.004 | 0.085 | 0.222 | 0.650 | <0.001 | 0.256 | <0.001 | 0.099 | <0.001 |
| T cells CD8 | 0.162 | <0.001 | 0.147 | 0.960 | 0.772 | 0.001 | 0.707 | <0.001 | 0.015 | 0.045 |
| T cells CD4 naive | 0.535 | 0.161 | 0.553 | 0.548 | 0.193 | 0.800 | 0.067 | 0.004 | 0.098 | 0.016 |
| T cells CD4 memory resting | 0.297 | 0.042 | 0.113 | 0.944 | 0.638 | 0.044 | 0.234 | 0.123 | 0.025 | <0.001 |
| T cells CD4 memory activated | 0.467 | 0.620 | 0.204 | 0.888 | 0.455 | 0.007 | 0.614 | 0.136 | 0.734 | 0.808 |
| T cells follicular helper | 0.543 | 0.368 | 0.879 | 0.146 | 0.525 | 0.216 | 0.139 | 0.893 | 0.175 | 0.321 |
| T cells regulatory | 0.155 | 0.005 | 0.359 | 0.236 | 0.106 | <0.001 | 0.006 | 0.054 | <0.001 | <0.001 |
| T cells gamma delta | 0.175 | 0.157 | 0.752 | 0.367 | 0.436 | <0.001 | 0.144 | 0.583 | 0.561 | 0.281 |
| NK cells resting | 0.039 | 0.021 | 0.701 | 0.680 | 0.448 | 0.762 | 0.500 | 0.157 | 0.561 | 0.008 |
| NK cells activated | 0.489 | 0.204 | 0.633 | 0.073 | 0.007 | 0.457 | 0.560 | <0.001 | 0.594 | 0.204 |
| Monocytes | 0.618 | 0.309 | 0.390 | 0.124 | 0.503 | <0.001 | 0.446 | 0.009 | 0.005 | <0.001 |
| Macrophages M0 | 0.068 | 0.313 | 0.991 | 0.963 | 0.161 | 0.926 | 0.569 | <0.001 | 0.002 | <0.001 |
| Macrophages M1 | 0.170 | 0.546 | 0.583 | 0.711 | 0.742 | <0.001 | 0.372 | 0.156 | 0.007 | <0.001 |
| Macrophages M2 | 0.060 | 0.373 | 0.213 | 0.235 | 0.785 | 0.049 | 0.479 | <0.001 | 0.979 | 0.051 |
| Dendritic cells resting | 0.449 | 0.445 | 0.384 | 0.505 | 0.700 | 0.336 | 0.747 | 0.088 | 0.103 | 0.897 |
| Dendritic cells activated | 0.201 | 0.850 | 0.138 | 0.504 | 0.195 | 0.049 | 0.185 | 0.434 | 0.332 | 0.580 |
| Mast cells resting | 0.628 | 0.263 | 0.869 | 0.467 | 0.158 | 0.632 | 0.531 | 0.638 | 0.735 | 0.026 |
| Mast cells activated | 0.939 | 0.411 | 0.812 | 0.915 | 0.105 | 0.570 | 0.022 | 0.270 | 0.755 | 0.003 |
| Eosinophils | 0.584 | 0.577 | 0.453 | 0.359 | 0.816 | 0.002 | 0.207 | 0.049 | 0.120 | 0.004 |
| Neutrophis | 0.765 | 0.134 | 0.230 | 0.589 | 0.516 | 0.994 | 0.301 | 0.106 | 0.377 | 0.7829 |
